# Supplementary material for: OPUS-DSD: deep structural disentanglement for cryo-EM single-particle analysis
Source: Nat Methods. 2023 Oct 9;20(11):1729–38. doi: 10.1038/s41592-023-02031-6 (PMC10630141; doi:10.1038/s41592-023-02031-6)
Supplement: Supplementary file 1 — Supplementary Table 1 and Fig. 1. [file 41592_2023_2031_MOESM1_ESM.pdf]

nature methods

Article

https://doi.org/10.1038/s41592-023-02031-6

OPUS-DSD: deep structural disentanglement for cryo-EM single-particle analysis

In the format provided by the authors and unedited

Supplementary Table 1. Cryo-EM data collection, refinement and validation statistics of NEXT complex

	#1 NEXT Complex<br>224,354 particles	#2 NEXT Complex<br>84,530 particles
**Data collection and processing**		
Magnification	81,000	81,000
Voltage (kV)	300	300
Electron exposure (e–/Å^2)	50	50
Defocus range (μm)	-1.0~-3.0	-1.0~-3.0
Pixel size (Å)	1.1	1.1
Symmetry imposed	C1	C1
Initial particle images (no.)	773,994	224,354
Final particle images (no.)	224,354	84,530
Map resolution (Å)	5.59	4.39
FSC threshold	0.143	0.143
Map resolution range (Å)		
Overall map	5.59	4.39
Focused Refinement ZCCHC8	4.76	4.57
**Refinement**	N/A	
Initial model used (PDB code)		AlphaFold2
Model resolution (Å).		4.39
FSC threshold		0.143
Model resolution range (Å)		
Overall Map		4.39
Focused Refinement ZCCHC8		4.57
Map sharpening *B* factor (Å^2)		-121
Model composition		
Non-hydrogen atoms		10887
Protein residues		1356
Ligands.		N/A
B factors (Å^2)		
Protein.		198.74
Ligand.		N/A
R.m.s. deviations		
Bond lengths (Å)		0.005
Bond angles (°)		0.718
Validation		
MolProbity score.		3.07
Clashscore		112.13
Poor rotamers (%)		0
Ramachandran plot		
Favored (%)		89.7
Allowed (%)		10.0
Disallowed (%)		0.3

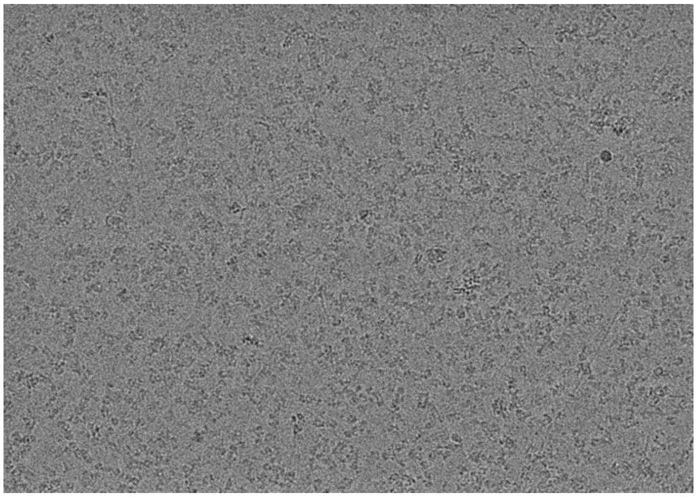

Supplementary Figure 1. A representative micrograph for NEXT complex.
