# Supplementary material for: OPUS-DSD: deep structural disentanglement for cryo-EM single-particle analysis
Source: Nat Methods. 2023 Oct 9;20(11):1729–38. doi: 10.1038/s41592-023-02031-6 (PMC10630141; doi:10.1038/s41592-023-02031-6)
Supplement: Supplementary file 2 — Reporting Summary [file 41592_2023_2031_MOESM2_ESM.pdf]

Corresponding author(s): Jianpeng Ma

Last updated by author(s): Jul 18, 2023

Reporting Summary

Nature Portfolio wishes to improve the reproducibility of the work that we publish. This form provides structure for consistency and transparency in reporting. For further information on Nature Portfolio policies, see our Editorial Policies and the Editorial Policy Checklist.

Statistics

For all statistical analyses, confirm that the following items are present in the figure legend, table legend, main text, or Methods section.

n/a	Confirmed	
☐	☒	The exact sample size (*n*) for each experimental group/condition, given as a discrete number and unit of measurement
☒	☐	A statement on whether measurements were taken from distinct samples or whether the same sample was measured repeatedly
☒	☐	The statistical test(s) used AND whether they are one- or two-sided<br>*Only common tests should be described solely by name; describe more complex techniques in the Methods section.*
☒	☐	A description of all covariates tested
☒	☐	A description of any assumptions or corrections, such as tests of normality and adjustment for multiple comparisons
☐	☒	A full description of the statistical parameters including central tendency (e.g. means) or other basic estimates (e.g. regression coefficient) AND variation (e.g. standard deviation) or associated estimates of uncertainty (e.g. confidence intervals)
☒	☐	For null hypothesis testing, the test statistic (e.g. *F*, *t*, *r*) with confidence intervals, effect sizes, degrees of freedom and *P* value noted<br>*Give P values as exact values whenever suitable.*
☐	☒	For Bayesian analysis, information on the choice of priors and Markov chain Monte Carlo settings
☒	☐	For hierarchical and complex designs, identification of the appropriate level for tests and full reporting of outcomes
☒	☐	Estimates of effect sizes (e.g. Cohen's *d*, Pearson's *r*), indicating how they were calculated

Our web collection on statistics for biologists contains articles on many of the points above.

Software and code

Policy information about availability of computer code

Data collection	Our data for NEXT complex was collected by SerialEM 4.0.
Data analysis	The following tools were used; KMeans clustering algorithm and principal component analysis in scikit-learn 1.3.0, Chimera 1.16, ChimeraX 1.6.1, cryoSPARC v2.4, Relion 3.0.8, umap v0.4.1, python 3.8, pytorch 1.10, cryoDRGN v1.0.0, and OPUS-DSD which is available at https://doi.org/10.24433/CO.3046690.v1 and https://github.com/alncat/opusDSD.

For manuscripts utilizing custom algorithms or software that are central to the research but not yet described in published literature, software must be made available to editors and reviewers. We strongly encourage code deposition in a community repository (e.g. GitHub). See the Nature Portfolio guidelines for submitting code & software for further information.

Data

Policy information about availability of data

All manuscripts must include a data availability statement. This statement should provide the following information, where applicable:

- Accession codes, unique identifiers, or web links for publicly available datasets
- A description of any restrictions on data availability
- For clinical datasets or third party data, please ensure that the statement adheres to our policy

We used the following publicly available datasets: EMPIAR-10180 (structure of a pre-catalytic spliceosome), EMPIAR-10028 (cryo-EM structure of a Plasmodium falciparum 80S ribosome bound to the anti-protozoan drug emetine) and EMPIAR-10002 (S.cerevisiae 80S ribosome direct electron detector dataset). Synthetic

and real NEXT datasets were deposited in Zenodo at https://doi.org/10.5281/zenodo.7748967. Trained models and heterogeneity analysis results are deposited at https://doi.org/10.5281/zenodo.8143779 .

Human research participants

Policy information about studies involving human research participants and Sex and Gender in Research.

Reporting on sex and gender	N/A
Population characteristics	N/A
Recruitment	N/A
Ethics oversight	N/A

Note that full information on the approval of the study protocol must also be provided in the manuscript.

Field-specific reporting

Please select the one below that is the best fit for your research. If you are not sure, read the appropriate sections before making your selection.

☒ Life sciences ☐ Behavioural & social sciences ☐ Ecological, evolutionary & environmental sciences

For a reference copy of the document with all sections, see nature.com/documents/nr-reporting-summary-flat.pdf

Life sciences study design

All studies must disclose on these points even when the disclosure is negative.

Sample size	For publicly available dataset used in this work, the sample size is as it is provided. For synthetic dataset generated in this work, the sample size is 64k, which is of comparable size to previous studies. For example, cryoDRGN1.0 generates a synthetic dataset with 50k particles. For NEXT complex dataset collected by our own group, the sample size is 224k. The number of particles of our NEXT complex dataset is also comparable to another published research of NEXT complex. In "Structural basis for RNA surveillance by the human nuclear exosome targeting (NEXT) complex" by M. Puno et.al (DOI:https://doi.org/10.1016/j.cell.2022.04.016) , 270k particles were used for 3D refinement.
Data exclusions	During training, part of data is randomly excluded from analysis and serves to validate our model. This is a standard technique known as cross-validation in machine learning.
Replication	The parameter settings for experiments are detailed in Methods section. We deposited models and codes on codeocean for replication with doi https://doi.org/10.24433/CO.3046690.v1 .
Randomization	Neural Networks are trained starting from random initialization weights and the dataset is randomly permuted at each iteration of training. The 3D refinement is also performed with random split of dataset. The training and validation sets are also randomly split.
Blinding	Researchers are blinded to expected results as the study is based on unsupervised learning.

Reporting for specific materials, systems and methods

We require information from authors about some types of materials, experimental systems and methods used in many studies. Here, indicate whether each material, system or method listed is relevant to your study. If you are not sure if a list item applies to your research, read the appropriate section before selecting a response.

Materials & experimental systems

n/a	Involved in the study	
☒	☐	Antibodies
☒	☐	Eukaryotic cell lines
☒	☐	Palaeontology and archaeology
☒	☐	Animals and other organisms
☒	☐	Clinical data
☒	☐	Dual use research of concern

Methods

n/a	Involved in the study	
☒	☐	ChIP-seq
☒	☐	Flow cytometry
☒	☐	MRI-based neuroimaging
